# Correction to “Realizing precision medicine in chronic lymphocytic leukemia: Remaining challenges and potential opportunities”

**DOI:** 10.1002/hem3.70249

**Published:** 2025-11-05

Stamatopoulos K, Pavlova S, Al‐Sawaf O, et al. Realizing precision medicine in chronic lymphocytic leukemia: remaining challenges and potential opportunities. *HemaSphere*. 2024;8(7):e113. doi:10.1002/hem3.113

A funder was incorrectly acknowledged in the original article. In the funding section, the sentence ‘…by the Italian Ministry of Health, grant PNRR‐MAD‐2022‐12376441 (Paolo Ghia) “Leukemic cell and microenvironment interactions as the culprit of chronicity in CLL” and grant PNRR‐MAD‐2022‐12375673 (Gianluca Gaidano) (Next Generation EU, M6/C2_CALL2022)…’ should have read ‘…by the European Union—Next Generation EU—NRRP M6C2—Investment 2.1 Enhancement and strengthening of biomedical research in the NHS—grant PNRR‐MAD‐2022‐12376441 (Paolo Ghia) cup master C43C22001280007 and grant PNRR‐MAD‐2022‐12375673 (Gianluca Gaidano) cup master J53C22004080001….’

The original article has been updated. We apologize for this error.

